# Plasma exosomal microRNA profiles associated with agitation symptoms in schizophrenia

**DOI:** 10.1186/s12888-026-08459-2

**Published:** 2026-07-30

**Authors:** Yucheng Ling, Yifan Wang, Ruiqiu Zhu, Yilin Tang, Ran Tao, Yantong Li, Yuxiu Sui

**Affiliations:** Department of Psychiatry, The Affiliated Brain Hospital of Nanjing Medical University, Nanjing, Jiangsu 210029 China

**Keywords:** Schizophrenia, Agitation, Exosomes, MicroRNAs

## Abstract

**Background:**

Inflammatory dysregulation and synaptic dysfunction have been implicated in the pathophysiology of agitation in schizophrenia (SZ), but the underlying molecular mechanisms remain unclear. Plasma exosomal microRNAs (miRNAs) are stable circulating molecules that may reflect disease-related molecular alterations associated with schizophrenia. This study aimed to investigate plasma exosomal miRNA expression profiles and peripheral inflammatory factors in schizophrenia patients with agitation, and to identify potential biomarkers for agitation symptoms.

**Methods:**

In this study, plasma exosomal miRNA expression profiles and peripheral inflammatory factors were compared between schizophrenia patients with agitation assessed within 24 h of hospital admission using PANSS-EC (SZA group, *n* = 35) and those without agitation (SZN group, *n* = 29). Differentially expressed plasma exosomal miRNAs were identified and subjected to GO and KEGG enrichment analyses. Peripheral inflammatory factors, including cytokine levels, neutrophil counts, and neutrophil-to-albumin ratio (NAR), were also measured. Partial correlation analyses (adjusting for age, sex, BMI, chlorpromazine equivalent, and diazepam equivalent) with Bonferroni correction were performed. A binary logistic regression (LR) model with forward stepwise selection (fixed covariates: age, sex, BMI, chlorpromazine equivalent, and diazepam equivalent) was constructed to identify discriminating factors, followed by receiver operating characteristic (ROC) curve analysis and five‑fold cross‑validation.

**Results:**

A total of 18 differentially expressed plasma exosomal miRNAs (15 upregulated, 3 downregulated) were identified in the SZA group. Target genes of upregulated miRNAs were enriched in MAPK and Ras signaling pathways, as well as cholinergic synapse and efferocytosis. Conversely, target genes of the downregulated miRNAs were specifically enriched in glutamatergic synapse. NAR showed a trend toward elevation in the SZA group (uncorrected *p* = 0.004, Bonferroni‑adjusted *p* = 0.088), while no significant differences in cytokine levels were observed. Partial correlation analysis revealed that in the SZA group, IL‑12p70 was positively correlated with miR‑3620‑5p (*r* = 0.893, adjusted *p* < 0.001) and miR‑17‑3p (*r* = 0.768, adjusted *p* = 0.028). Multivariable logistic regression (including fixed covariates: age, sex, BMI, chlorpromazine equivalent, diazepam equivalent) retained NAR (OR = 3.778, *p* = 0.006) and miR‑543 (OR = 0.162, *p* = 0.026). The combined model (NAR + miR‑543 + fixed covariates) achieved an AUC of 0.812, outperforming single‑marker models (NAR AUC = 0.752, miR‑543 AUC = 0.715). Five‑fold cross‑validation yielded a mean AUC of 0.795 (95% CI 0.675–0.916), with sensitivity 80.1 ± 16.5%, specificity 79.5 ± 26.1%, and accuracy 77.8 ± 8.9%.

**Conclusion:**

The findings suggest that schizophrenia patients with agitation exhibit distinct plasma exosomal miRNA expression profiles and peripheral inflammatory alterations compared to those without agitation. These findings also imply potential involvement of inflammation-associated signaling and synaptic pathways in the biological mechanisms underlying agitation in schizophrenia. Furthermore, the combination of NAR and miR-543 may serve as a potential biomarker for agitation assessment in schizophrenia, offering new insights into the biological mechanisms underlying this clinically challenging symptom.

**Supplementary Information:**

The online version contains supplementary material available at https://doi.org/10.1186/s12888-026-08459-2.

## Introduction

Schizophrenia is a complex psychiatric disorder characterized by a broad range of clinical features, including positive symptoms, negative symptoms, cognitive deficits, agitation and affective symptoms [1]. Agitation is one of the vital symptoms of schizophrenia (SZ), defined as a state of motor restlessness accompanied by mental tension, characterized by restlessness, irritability, anxiety, and excessive movement or speech without a specific purpose [2]. As a major cause of hospitalization in patients with schizophrenia [3], agitation occurs with an incidence ranging from 15% to 53% [4]. Clinically, agitation is considered as a continuum that extends from inner tension and restlessness to further aggressive or violent behavior, with no clear boundaries between these states [5]. Such uncontrolled behavior poses serious risks to patients’ families, healthcare providers, and society [6]. In addition, agitation is also considered to be associated with more severe positive symptoms [7] and poorer prognosis [8]. Therefore, it is essential to further clarify the biological mechanisms underlying agitation in schizophrenia.

Synaptic dysfunction is a key contributor in schizophrenia. Synaptic abnormalities, including altered expression of synaptic proteins (e.g., postsynaptic density protein 95 (PSD95), synaptophysin), reduced dendritic spine density, and impaired synaptic plasticity, have been consistently observed in postmortem brain tissues of schizophrenia patients [9, 10]. These alterations are particularly pronounced in brain regions implicated in emotion and behavior regulation, such as the prefrontal cortex and hippocampus [11]. Notably, glutamatergic dysfunction [12] and NMDA receptor abnormalities in GABAergic synapses [13] have been documented in these regions in schizophrenia. Despite synaptic dysfunction being well established in schizophrenia, studies directly examining synaptic alterations in schizophrenia patients with agitation symptoms remain limited. Inflammatory dysregulation is another important pathophysiological mechanism underlying agitation in schizophrenia. Clinical studies indicate that patients with schizophrenia exhibiting agitation symptoms demonstrate elevated levels of multiple pro-inflammatory factors in their peripheral blood. A study involving 119 schizophrenia patients found that the high-agitation group had higher levels of the pro-inflammatory cytokine interleukin-6 (IL-6) than the low-agitation group. Markers associated with M1-type macrophages and indicators of the immune-inflammatory response system also showed an upward trend [14]. Elevated tumor necrosis factor-α levels (TNF-α) [15] and C-reactive protein (CRP) [16] levels have also been reported in schizophrenia patients with agitation. Although direct evidence in patients with agitation remains limited, central neuroinflammation has been associated with overall symptom severity in schizophrenia. Neuropathological and neuroimaging studies have revealed activated microglia and regional gray matter volume loss in the prefrontal cortex [17]. In a pro-inflammatory environment, activated microglia can promote excessive synaptic pruning, particularly of glutamatergic synapses, resulting in excitation/inhibition imbalance [18]. Through microglia-mediated synaptic remodeling, neuroinflammation may further disrupt glutamatergic neurotransmission and neural circuit function, thereby providing a mechanistic link between inflammatory dysregulation and synaptic abnormalities. Such alterations are particularly evident in the prefrontal [19, 20] and cingulate cortex [21], brain regions critical for emotional regulation and impulse control, and may partially underlie the pathophysiological mechanisms of agitation in schizophrenia. Collectively, while synaptic dysfunction and inflammatory dysregulation are well-established features of schizophrenia, their specific contributions to agitation remain incompletely understood and warrant further investigation.

Given the difficulty in obtaining brain tissue and cerebrospinal fluid samples, it is challenging to investigate structural and functional abnormalities in synapses. Due to the presence of the blood-brain barrier (BBB), peripheral and central inflammatory levels are not perfectly correlated. Indeed, only weak correlations have been observed for IL-6 and TNF-α [22]. A review of patients with schizophrenia spectrum disorders revealed a dissociation between central and peripheral immune markers. Specifically, individuals exhibiting signs of central immune dysfunction, such as positive oligoclonal bands and elevated albumin quotient (QAlb) levels in cerebrospinal fluid (CSF), did not show corresponding elevations in peripheral blood inflammatory markers like CRP or neutrophil counts [23]. Moreover, peripheral cytokine concentrations are influenced by multiple biological and pre-analytical factors, including antipsychotic treatment, circadian variation, fasting status, sample collection, and processing conditions, which may further affect their reproducibility and interpretation [24, 25]. These findings suggested the limitations of using peripheral blood markers to reflect central immune status. Therefore, there is a need for biomarkers that can capture disease-related molecular alterations beyond conventional peripheral inflammatory markers.

Exosomes, as carriers discovered in recent years, have double-layered lipid vesicles with a diameter of approximately 150 nm that can cross the BBB and be detected from peripheral blood [26]. Exosomes are widely present in bodily fluids, including blood [27], urine [28], and cerebrospinal fluid [29], and can transport and deliver substances such as proteins, lipids, and RNA [30]. Unlike free circulating miRNAs, which are vulnerable to pre-analytical factors such as temperature and storage duration [31, 32], exosomes package their cargo within a protective lipid bilayer, conferring greater stability and resistance to degradation in the peripheral circulation [33]. Consistent with these advantages, accumulating evidence has demonstrated that peripheral blood exosomes contain disease-related molecular cargo associated with schizophrenia. For instance, inflammatory-related miRNAs from schizophrenia patients, such as the miR-15/107 family and miR-103, have been reported to exhibit increased expression in brain tissue [34, 35], while similar dysregulation has also been observed in plasma exosomes [36]. These findings suggest that plasma exosomal miRNAs may capture disease-related molecular alterations associated with schizophrenia and therefore represent promising peripheral biomarkers. Furthermore, evidence suggests that exosomes also carry synaptic-related proteins, such as microtubule-associated protein 1B (MAP1B) [37] and dysbindin-1, which is encoded by the schizophrenia susceptibility gene Dystrobrevin Binding Protein 1(DTNBP1) [38]. To sum up, plasma exosomes carry molecular cargo associated with inflammatory and synaptic processes and may serve as peripheral biomarkers for investigating the biological mechanisms underlying agitation in schizophrenia.

Accordingly, using plasma exosomal miRNAs as biomarkers, this study aimed to investigate the expression profiles of plasma exosomal miRNAs in schizophrenia patients with and without agitation symptoms and identify predictive exosomal miRNAs associated with agitation. We hypothesized that agitation is accompanied by distinct alterations in exosomal miRNAs related to inflammatory and synaptic processes. By exploring these post-transcriptional molecular changes, this study sought to provide insights into the biological mechanisms underlying agitation in schizophrenia and to identify potential biomarkers for agitation.

## Materials and methods

### Participants

Sixty-four SZ patients were recruited from Nanjing Brain Hospital (NBH) between 2025 and 2026.The inclusion criteria were as follows: (i) age between 18 and 55 years old; (ii) meeting the Diagnostic and Statistical Manual of Mental Disorders, fifth edition (DSM-5) criteria for schizophrenia, as confirmed by two experienced psychiatrists; (iii) elementary school education and above; (iv) without other psychiatric disorders; (v) without recent infection, brain injury, metabolic diseases or autoimmune diseases; (vi) without current or past use of tobacco, alcohol, or other psychoactive substances. The antipsychotic medications taken by schizophrenia patients upon admission and before blood sampling and PANSS assessment were converted to chlorpromazine equivalent [39], and any benzodiazepines administered were converted to diazepam equivalent [40]. All participants provided written informed consent, and the study was approved by the Ethics Committee of Nanjing Brain Hospital. A summary of demographic and clinical characteristics for all the participants was shown in Table 1.

Positive and Negative Syndrome Scale (PANSS) assessments were administered within 24 h of admission. The agitation symptoms were evaluated by the PANSS excitatory component (PANSS-EC), which included excitement (P4), hostility (P7), tension (G4), uncooperativeness (G8), and poor impulse control (G14). The PANSS-EC total score ≥ 14, with one or more items scoring ≥ 4, indicates the presence of agitation [15, 41, 42]. According to the criteria, the SZ patients in this study were divided into non-agitated (SZN, *n* = 29) and agitated (SZA, *n* = 35) subgroups.

### Plasma collection

Within 24 h of admission, fasting blood samples were drawn from the anterior elbow vein. All blood samples were collected using EDTA-K2 anticoagulant tubes or gel-separator tubes with clotting agents, depending on the testing purpose, and processed within 4 h at 4 °C.

The concentration of plasma cytokines, including 10 selected pro-inflammatory cytokines (IL-2, IL-5, IL-6, IL-8, IL-1β, IL-12p70, IL-17, IFN-α, IFN-γ, and TNF-α) and 2 anti-inflammatory cytokines (IL-4 and IL-10), was quantified using the enzyme-linked immunosorbent assay (ELISA) method. Fasting venous blood samples were collected from all subjects within 24 h of admission and transferred to EDTA anticoagulant tubes. The samples were then centrifuged (1000 g/min, 4 °C, 10 min) to obtain the supernatant, which was stored at − 80 °C for further analysis. For each cytokine, a 96-well ELISA plate was set up with blank wells, standard wells, and sample wells, as well as duplicate wells. The optical density of the samples was determined using a standard curve based on the measurement of standard samples. All samples were analyzed by two experienced technicians who were blinded to the patients’ identities and clinical information. The within-run and between-run coefficients of variation for all analyses were found to be less than 10%.

Neutrophil absolute count, lymphocyte absolute count, and monocyte absolute count were determined using flow cytometry. Platelet count was measured by impedance method. The neutrophil to lymphocyte ratio (NLR) was calculated by dividing the neutrophil absolute count by the lymphocyte absolute count. The neutrophil to monocyte ratio (NMR), platelet to lymphocyte ratio (PLR), and monocyte to lymphocyte ratio (MLR) were calculated similarly. Albumin concentration was measured using the bromocresol green method. The neutrophil to albumin ratio (NAR) was calculated by dividing the neutrophil count by the albumin concentration.

### Isolation of plasma exosomes

Blood samples were processed using a two-step centrifugation protocol. First, samples were centrifuged at 1900 g for 10 min, followed by collection of the supernatant plasma. The supernatant was then centrifuged at 3000 g for 15 min. After the second centrifugation, the resulting supernatant was collected and stored at − 80 °C until further processing for sequencing.

Plasma exosomes were isolated by ultracentrifugation. Frozen plasma samples were thawed at 37 °C and centrifuged at 2,000 × g for 30 min and subsequently at 10,000 × g for 45 min at 4 °C to remove cellular debris and larger vesicles. The resulting supernatant was filtered through a 0.45 μm membrane (Millipore, R6BA09493) and ultracentrifuged at 100,000 × g for 70 min at 4 °C. The exosome pellet was resuspended in 10 mL of pre-cooled 1× PBS (Sangon Biotech, E607008), washed once by ultracentrifugation under the same conditions, and finally resuspended in 100 µL of pre-cooled 1× PBS. The purified exosome suspension was subsequently characterized by transmission electron microscopy (TEM) and nanoparticle tracking analysis (NTA), whereas the remaining suspension was stored at − 80 °C.

### Characterization of exosomes

Exosome characterization was performed using transmission electron microscopy (TEM) and nanoparticle tracking analysis (NTA). For TEM analysis, purified exosome suspensions were mounted onto copper grids, negatively stained with uranyl acetate (Henan Xinyue, GZ02625-5), air-dried, and examined using a transmission electron microscope (Hitachi HT-7700) operated at 80 kV. For NTA analysis, cryopreserved exosome samples were thawed, diluted appropriately with 1× PBS, and analyzed using a ZetaVIEW nanoparticle tracking analyzer (Particle Metrix).

### RNA isolation

Total RNA was extracted from plasma exosomes using TRIzol^®^ LS Reagent (10296028, Thermo Fisher Scientific) according to the manufacturer’s instructions. Thawed exosome suspensions were lysed with TRIzol LS reagent and supplemented with miRNeasy Serum/Plasma Spike-In Control (219610, QIAGEN). Following chloroform extraction and centrifugation for phase separation, RNA was precipitated with isopropanol (YC-SJ06035, Sinopharm Group) and glycogen (R0551, Thermo Fisher Scientific), washed twice with pre-cooled 75% ethanol, and resuspended in RNase-free water (10977015, Thermo Fisher Scientific). RNA quality was assessed before library preparation, and the remaining RNA was stored at − 80 °C until further analysis.

### Library preparation

Small RNA libraries were constructed using the NEBNext^®^ Multiplex Small RNA Library Prep Set for Illumina (NEB, UK, #E7560S) according to the manufacturer’s instructions. Total RNA (100 ng–1 µg) extracted from plasma-derived exosomes was used as input material. Briefly, sequential 3′ and 5′ adaptor ligation, reverse transcription, PCR amplification, purification, and gel-based size selection were performed to generate miRNA libraries (140–160 bp). The purified libraries were resuspended in TE Buffer (NEB, UK) for subsequent quality control and sequencing.

### Library quality control and RNA sequencing

Library size distribution and concentration were evaluated using an Agilent 2100 Bioanalyzer. Libraries that met the quality control criteria (expected peak size of approximately 147 bp) were pooled at equimolar concentrations before sequencing on an Illumina NovaSeq 6000 platform using a paired-end 150 bp (PE150) strategy.

### Differential expression analysis

The quality of the raw sequencing data, including sequencing accuracy and base composition at each position, was assessed using FastQC (v0.11.8). Next, fastp (v0.19.5) was used to remove adapter sequences and low-quality reads, producing clean reads. Quality control of miRNA sequences was performed using blastn (v2.9.0). The filtered sequences were aligned to the reference genome using bowtie (v1.2.3), and miRNA expression levels (counts) were determined with miRDeep2 (v0.1.3) based on the alignment data. Differential expression analysis was carried out using the R package DESeq2 (v1.10.1), with the selection criteria of |log₂ fold change (FC)| ≥ 1 and P Adjust (after Benjamini-Hochberg correction) < 0.05. MiRanda (v3.3) and RNAhybrid were used to predict target genes for differentially expressed miRNAs, and the intersection of the results from both algorithms was retained for subsequent enrichment analysis. Enrichment analysis of target genes was performed using clusterProfiler (v2.4.2) on Gene Ontology (GO) categories, including biological process (BP), molecular function (MF), and cellular component (CC), as well as Kyoto Encyclopedia of Genes and Genomes (KEGG) pathways. Fisher’s exact test was used for enrichment analysis with a threshold of FDR < 0.05. (Benjamini‑Hochberg correction)

### Statistical analysis

#### Descriptive statistics

Data distribution normality was tested using the Shapiro-Wilk test. Normally distributed continuous variables are presented as mean ± standard deviation (SD) and compared between two groups using independent samples t-test. Non-normally distributed data are presented as median with (interquartile range, IQR) and compared with Mann–Whitney U tests. Categorical variables are presented as frequencies (%) and compared using chi-square tests. A two-tailed *p* < 0.05 was considered statistically significant.

#### Multiple comparison corrections

For differentially expressed plasma exosomal miRNAs, the Benjamini-Hochberg method was applied to control the false discovery rate (FDR). MiRNAs with adjusted p-values < 0.05 were considered significantly differentially expressed. These significantly differentially expressed miRNAs were subjected to GO functional enrichment and KEGG pathway enrichment analyses, and only pathways with Benjamini-Hochberg adjusted p values < 0.05 were discussed. For demographic and clinical data and peripheral blood inflammatory factors, the Bonferroni correction was applied for multiple comparisons. A corrected p-value < 0.05 was considered statistically significant.

#### Partial correlation analysis

Partial correlation analysis was performed to assess the associations between 22 peripheral blood inflammatory factors and 18 differentially expressed plasma exosomal miRNAs, with age, sex, body mass index (BMI), diazepam equivalent, and chlorpromazine equivalent as covariates. The p values from the correlation analyses were adjusted for multiple comparisons using the Bonferroni correction. Only correlations with a Bonferroni-corrected p-value < 0.05 were discussed.

#### Binary logistic regression for discriminant model

To identify key factors discriminating agitated from non-agitated schizophrenia patients, binary logistic regression was performed. The dependent variable was group status. The independent variables included: (1) fixed covariates (Model 1): age, sex (female as reference), BMI, diazepam equivalent, and chlorpromazine equivalent; (2) candidate variables: 22 peripheral blood immune‑inflammatory markers and 18 plasma exosomal miRNAs. Due to large differences in measurement scales, all candidate variables were Z‑score standardized before analysis.

Forward stepwise selection based on the likelihood ratio test was used for variable selection, with an entry criterion of *p* < 0.05. The fixed covariates were forced into the model. After the stepwise procedure, three candidate variables (PLR, NAR and miR-543) met the entry criterion and were retained in the model. Among them, two variables (NAR and miR-543) remained statistically significant (*p* < 0.05) in the final model. Since PLR was not statistically significant in the final model, the receiver operating characteristic analyses (ROC) included only NAR and miR-543, together with the fixed covariates. The area under the curve (AUC) with 95% confidence interval (CI) was calculated. All tests were two‑tailed, and a p‑value < 0.05 was considered statistically significant. SPSS version 27.0 was used for all analyses.

#### Cross‑validation

To assess the generalization ability of the final model, five‑fold cross‑validation was performed on the dataset of 64 samples. The dataset was randomly divided into 5 equal folds. In each iteration, one fold served as the validation set and the remaining four folds as the training set. For the training set, a logistic regression model was built with the two identified markers (NAR and miR-543) together with the fixed covariates (age, sex, BMI, diazepam equivalent, chlorpromazine equivalent) as independent variables, and the group status as the dependent variable. The predicted probability for each sample in the validation set was saved. This process was repeated five times, with each fold used exactly once as the validation set. For each validation set, the AUC was calculated based on the predicted probabilities and the actual group labels. The optimal cutoff was determined by maximizing the Youden index. Sensitivity, specificity, accuracy, and Youden index were then computed. The average AUC across the five folds was reported as the arithmetic mean.

## Result

### Demographic and clinical characteristics

Demographic and clinical characteristics are shown in Table 1. A total of 64 schizophrenia patients were included in this study, comprising 29 patients without agitation symptoms (SZN) and 35 patients with agitation symptoms (SZA). There were no significant differences in age, gender, BMI, duration of illness, age of onset or chlorpromazine equivalent, or diazepam equivalent between the two groups (all *p* > 0.05). Analysis revealed significant differences in several clinical scales: the SZA group had significantly higher PANSS-EC scores (t = 12.527, P Adjust < 0.001), PANSS total scores (t = 4.630, P Adjust < 0.001), PANSS positive subscale scores (t = 4.742, P Adjust < 0.001), and PANSS general psychopathology subscale scores (t = 3.304, P Adjust = 0.024) compared to the SZN group. No significant difference was found in the PANSS negative subscale scores between the groups (t = -0.95, P Adjust = 1).

**Table 1:** Demographic and clinical data of SZN and SZA

	SZN(*n* = 29)	SZA(*n* = 35)	t/X^2^/Z	*P* value	*P* Adjust
Age(year)	29.10(8.74)	29.63(8.28)	0.25	0.81^b^	1
Male (%)	31.0	45.7	1.436	0.23 ^c^	1
BMI (kg/m^2^)	22.51(4.23)	23.36(4.77)	0.75	0.458 ^b^	1
Duration of illness(year)	7.03(5.84)	8.67(6.73)	1.03	0.31 ^b^	1
Age of Onset(year)	22.17(7.55)	21.00(6.78)	0.65	0.52 ^b^	1
Chlorpromazine Equivalent	340.56(188.74)	344.82(187.69)	0.089	0.93 ^b^	1
Diazepam Equivalent ^a^	0.00(5.00)	4.00(5.00)	0.271	0.787 ^d^	1
PANSS total score	89.45(10.47)	103.20(12.84)	4.63	< 0.001***^b^	< 0.001***
PANSS-EC	8.93(2.70)	17.43(2.71)	12.53	< 0.001***^b^	< 0.001***
PANSS positive score	20.62(5.07)	25.86(3.75)	4.74	< 0.001***^b^	< 0.001***
PANSS Negative Score	26.69(5.53)	25.46(4.89)	-0.95	0.35^b^	1
PANSS General Score	38.07(4.94)	42.97(6.60)	3.30	0.002***^b^	0.024*

Data are presented as mean (SD) or median (IQR). N is sample size

a non-normal distribution data

b Independent samples T-test

c Chi-square test

d Mann-Whitney U test

* significant at *P* < 0.05.** significant at *P* < 0.005.***significant at *P* < 0.001. Using the Bonferroni correction to adjust P value. (P value multiplied by 12, capped at1.000)

* significant at P Adjust < 0.05. ** significant at P Adjust < 0.005. ***significant at P Adjust < 0.001. Abbreviation: SZN: non-agitated schizophrenia group, SZA: agitated schizophrenia group, BMI: body mass index, PANSS: positive and negative syndrome scale, PANSS-EC: Positive and Negative Syndrome Scale-Excited Component

**Fig. 1:**
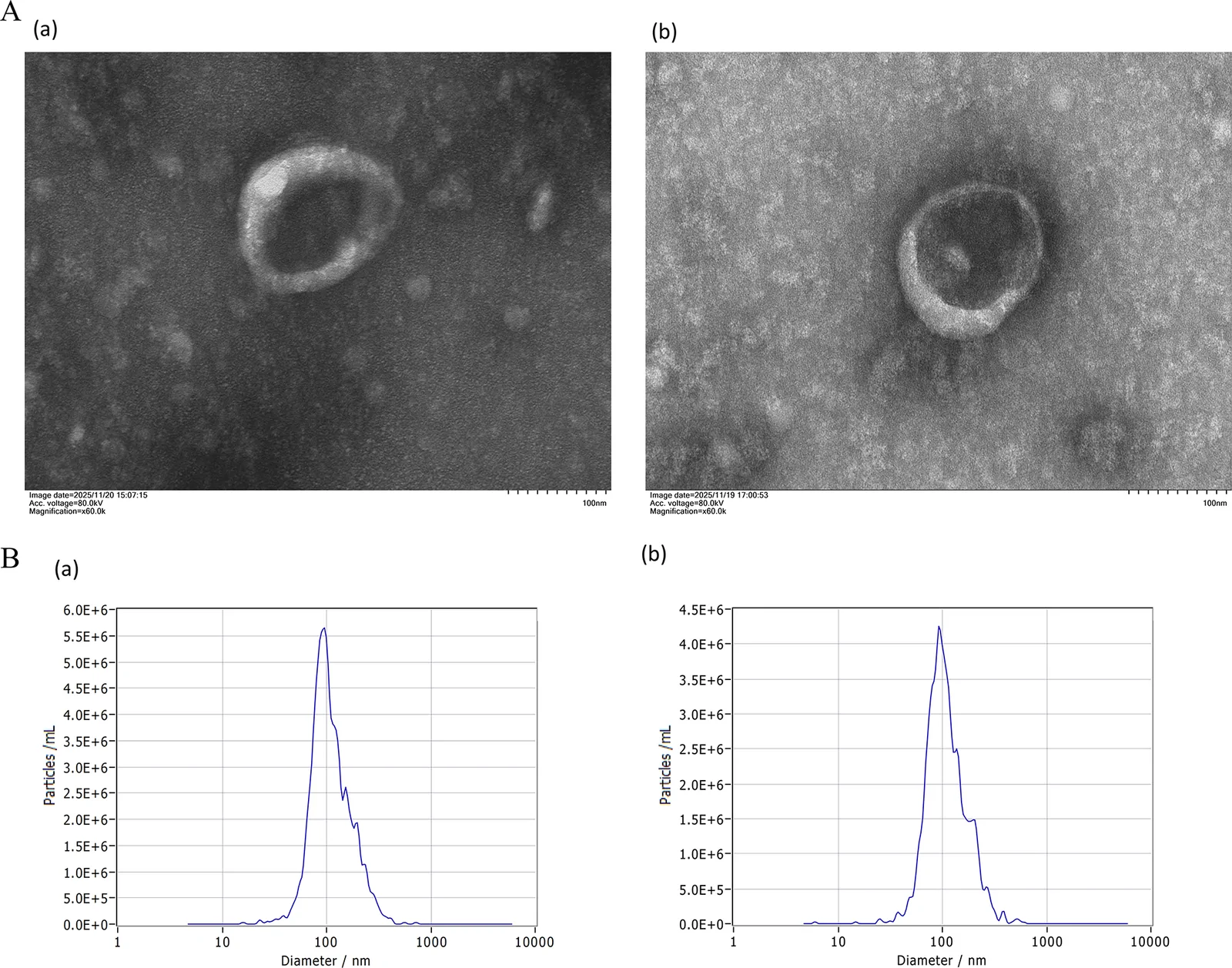
Characterization of total exosomes isolated from human plasma. **(A)** The typical morphological characteristics of isolated exosomes were detected by TEM. **(B)** The size distribution of plasma exosomes was determined by NTA. (**a**) SZA group, (**b**) SZN group. TEM: Transmission Electron Microscope, NTA: Nanoparticle Tracking Analysis

### Isolation and characterization of plasma exosomes

Exosomes were isolated from the plasma of SZA and SZN groups via ultracentrifugation. The isolated exosomes were then characterized by Transmission Electron Microscope (TEM) and Nanoparticle Tracking Analysis (NTA), as presented in Fig. 1. The isolated exosomes exhibited a typical cup-shaped morphology with a visible bilayer membrane under TEM (Fig. 1A). NTA revealed that the exosomes size were approximately 100 nm in both SZN and SZA group (Fig. 1B).

### Differential expression of miRNAs in SZA group

Plasma exosomal microRNA sequencing was performed on all participants (SZN = 29, SZA = 35). Following quality control and normalization, microRNAs with low expression levels were filtered out. MiRNA-seq found 1635 exosomal miRNAs (Supplementary Table 1). Differentially expressed miRNAs were defined as |Log2FoldChange| ≥1 and an adjusted p-value < 0.05 using the Benjamini-Hochberg correction. A total of 18 differentially expressed miRNAs were identified, including 15 upregulated and 3 downregulated miRNAs (Table 2).

**Table 2:** Differentially expressed microRNAs in SZA group

Differentially expressed miRNAs	Log2 Fold Change	*P* Adjust	Differentially expressed miRNAs	Log2 Fold Change	*P* Adjust
hsa-miR-3198	4.85	0.0047	hsa-miR-874-3p	1.73	0.0181
hsa-miR-10400-5p	4.03	0.0416	hsa-miR-200b-3p	1.71	0.0153
hsa-miR-6515-5p	3.96	4.0148 × 10^− 6^	hsa-miR-4508	1.58	0.0324
hsa-miR-6741-5p	3.60	1.1541 × 10^− 5^	hsa-miR-17-3p	1.57	0.0495
hsa-miR-6734-5p	3.47	1.4547 × 10^− 6^	hsa-miR-2110	1.52	0.0047
hsa-miR-3620-5p	3.13	0.0181	hsa-miR-1292-5p	1.46	0.0153
hsa-miR-671-5p	3.07	0.0047	hsa-miR-4488	-4.34	8.7932 × 10^− 10^
hsa-miR-296-3p	2.72	0.0153	hsa-miR-9-5p	-2.85	0.0002
hsa-miR-4665-5p	2.08	0.0194	hsa-miR-543	-1.14	0.0495

Significant differential expressed miRNAs were identified in the SZA group (*n* = 35) compared to SZN (*n* = 29) group. The miRNAs listed all have an adjusted P value < 0.05 (using Benjamini-Hochberg correction)

Abbreviation: SZN: non-agitated schizophrenia group, SZA: agitated schizophrenia group, miRNAs: microRNAs

### Target gene prediction and functional enrichment analysis of differentially expressed miRNAs

**Fig. 2:**
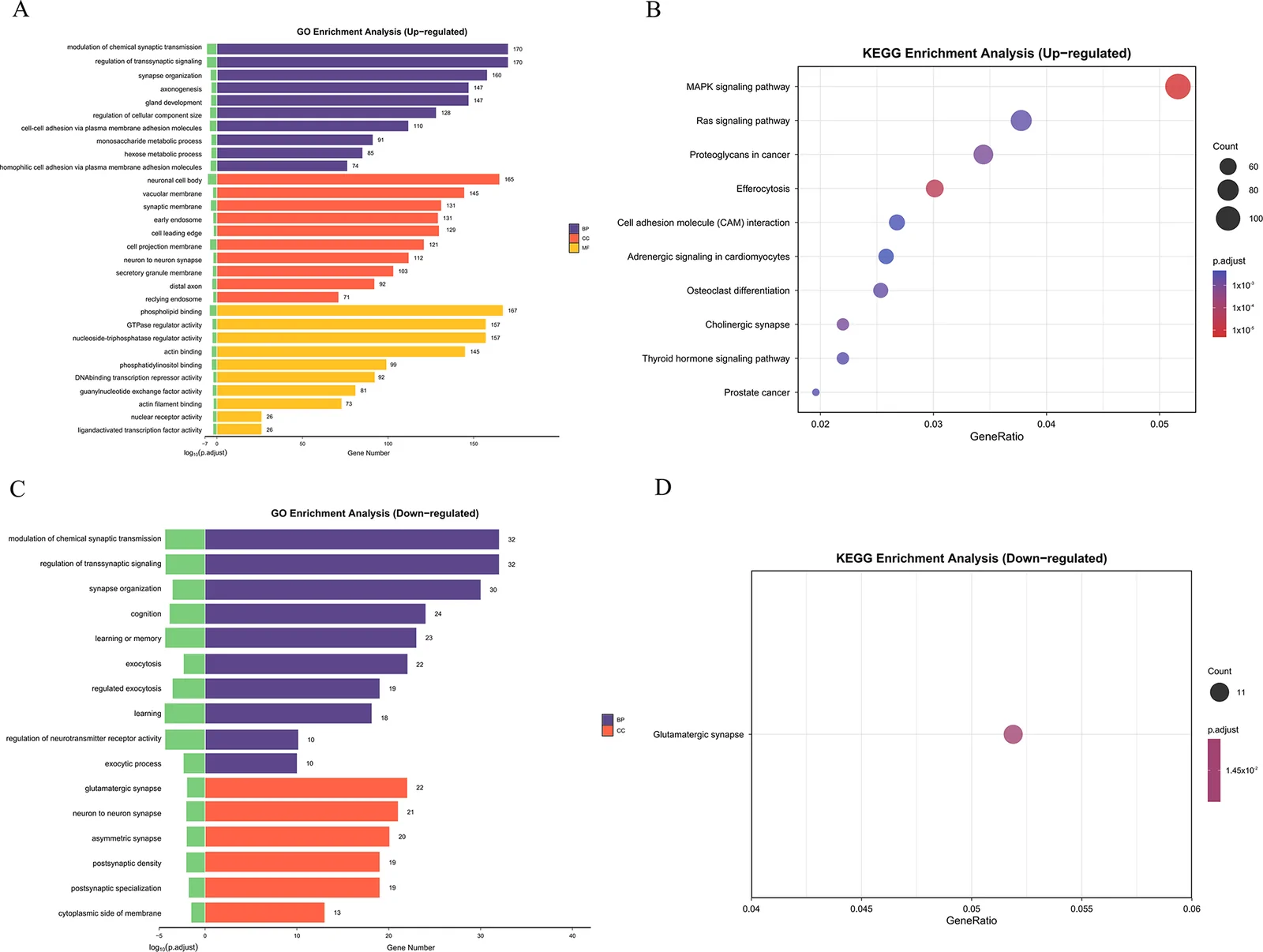
Functional analysis of differential expression miRNAs target genes in SZA group. **(A-B)**. GO enrichment analysis and KEGG pathway analysis of the target genes predicted by upregulated miRNAs in SZA group. **(C-D).** GO enrichment analysis and KEGG pathway analysis of the target genes predicted by downregulated miRNAs in SZA group. miRNA: microRNA, GO: Gene Ontology, KEGG: Kyoto Encyclopedia of Genes and Genomes, BP: Biological Process, CC: Cellular Component, MF: Molecular Function

The predicted target genes of the 18 differentially expressed miRNAs were subjected to GO and KEGG pathway enrichment analyses (both after Benjamini-Hochberg correction). Figure 2 shows the most enriched GO and KEGG elements. The results of the other pathway enrichment analyses can be found in Supplementary Material 2.

For the upregulated group, biological processes were enriched in modulation of chemical synaptic transmission, regulation of trans-synaptic signaling, synapse organization, axonogenesis and gland development. For the cellular component, these target genes were predominantly localized to neuronal cell body, vacuolar membrane, synaptic membrane, cell leading edge and endosome (recycling and early). Molecular functions were significantly enriched in phospholipid binding, GTPase regulator activity, nucleoside-triphosphatase regulator activity, actin binding and phosphatidylinositol binding.

In contrast, for the downregulated group, GO analysis focused on synapse and exocytosis. Biological processes were significantly associated with modulation of chemical synaptic transmission, regulation of transsynaptic signaling, synapse organization and exocytosis. Cellular components were chiefly localized to synapse, including the glutamatergic synapse, neuron to neuron synapse, asymmetric synapse, postsynaptic density, and postsynaptic specialization.

KEGG pathway analysis further elucidated their functional divergence. Target genes of the upregulated group were significantly involved in the MAPK signaling pathway, Ras signaling pathway, proteoglycans in cancer and efferocytosis. Conversely, target genes of the downregulated group were only enriched in glutamatergic synapse.

### Peripheral immune inflammatory characteristics in the SZA group

Peripheral blood inflammatory factors are presented in Table 3. A total of 64 schizophrenia patients were included in the analysis, comprising 29 patients without agitation symptoms (SZN) and 35 patients with agitation symptoms (SZA).

**Fig. 3:**
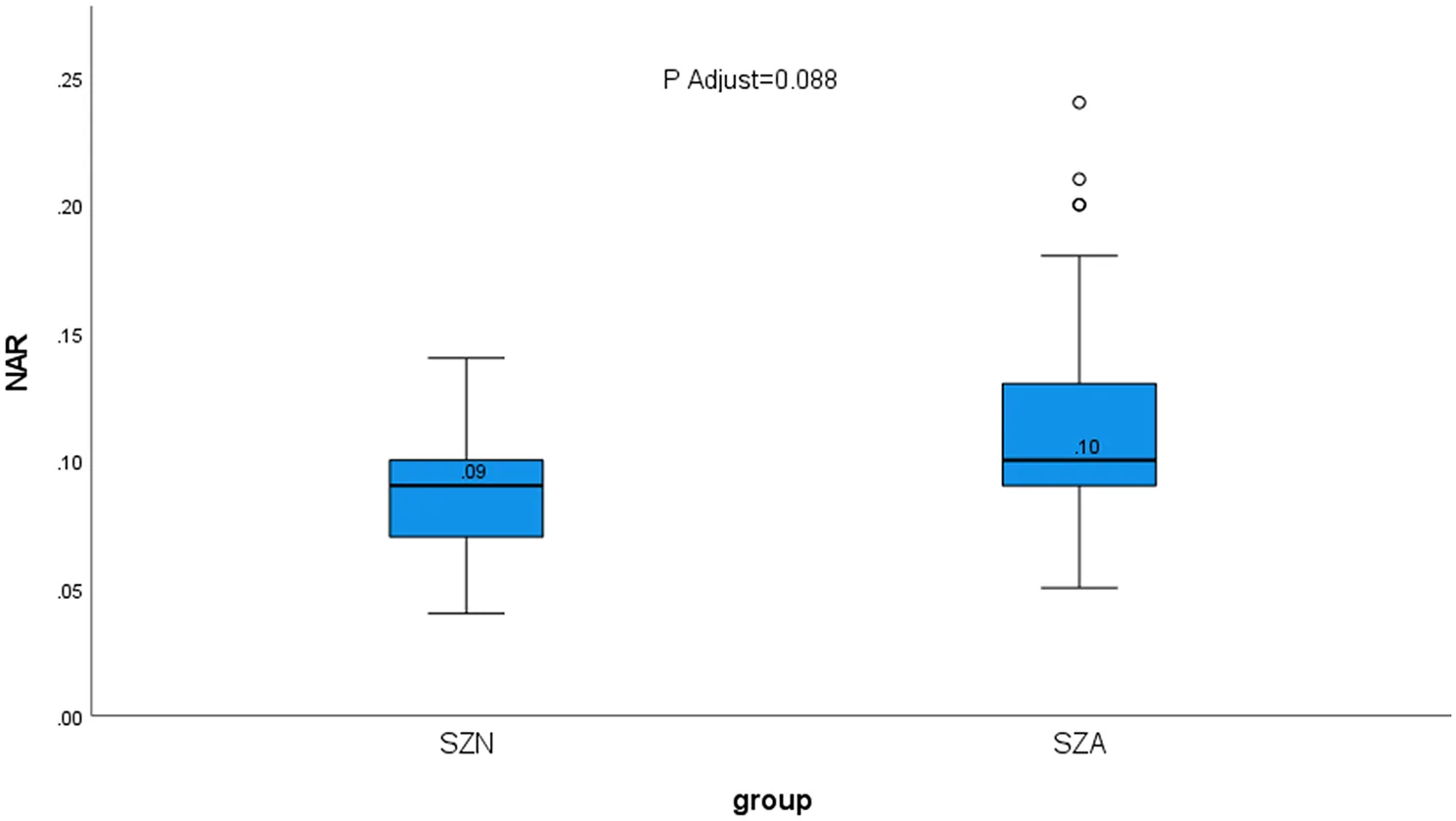
Boxplot of NAR in SZN and SZA group. The horizontal line inside each box represents the median, the box boundaries denote the interquartile range (IQR) and the whiskers extend to the minimum and maximum values within 1.5×IQR. Abbreviation: SZN: non-agitated schizophrenia group, SZA: agitated schizophrenia group, NAR: neutrophil-to-albumin ratio

In uncorrected analysis, the SZA group showed a trend toward higher NAR levels (Z = 2.81, *p* = 0.004) compared with the SZN group, but this difference did not pass Bonferroni correction (adjusted *p* = 0.088, Fig. 3), suggesting only a statistical trend. No significant differences were found between the two groups in any other peripheral blood markers (all adjusted *p* > 0.05), including neutrophil counts, interleukins (IL-2, IL-4, IL-5, IL-6, IL-8, IL-10, IL-17, IL-1β, IL-12p70), interferons (IFN-α, IFN-γ), tumor necrosis factor‑α (TNF-α), lymphocytes, monocytes, platelets, albumin, NLR, NMR, PLR, and MLR.

**Table 3:** Comparison of peripheral blood inflammatory factors between SZA and SZN group

	SZN(*n* = 29)	SZA(*n* = 35)	t/Z	*P* value	*P* Adjust
IL-2(pg/mL)	1.99(0.52)	1.79(0.43)	-1.47	0.149^a^	1
IL-4(pg/mL)	1.86(0.17)	1.82(0.17)	-0.86	0.396^a^	1
IL-5(pg/mL)	1.96(0.44)	1.89(0.30)	-0.64	0.524^a^	1
IL-6(pg/mL)	3.50(2.66)	4.26(2.68)	0.96	0.344^a^	1
IL-8(pg/mL)	8.14(11.33)	7.21(8.69)	-0.32	0.754^a^	1
IL-10(pg/mL)	1.45(0.38)	1.35(0.30)	-1.00	0.323^a^	1
IL-17(pg/mL)	2.81(1.45)	2.40(0.59)	0.474	0.635^b^	1
IL-1β(pg/mL)	3.11(1.37)	2.91(1.45)	-0.46	0.649^a^	1
IL-12p70(pg/mL)	1.69(0.19)	1.81(0.62)	0.80	0.429^a^	1
IFN-α(pg/mL)	1.53(0.40)	1.48(0.32)	-0.43	0.673^a^	1
IFN-γ(pg/mL)	3.93(6.03)	2.90(1.96)	-0.81	0.422^a^	1
TNF-α(pg/mL)	2.37(0.31)	2.46(0.72)	0.51	0.609^a^	1
Neutrophils(10^9^/L)	3.49(0.95)	4.67(1.84)	2.778	0.005^b^	0.110
Lymphocytes(10^9^/L)	1.86(0.57)	2.10(0.79)	1.36	0.178^a^	1
Monocytes(10^9^/L)	0.47(0.16)	0.50(0.15)	0.71	0.483^a^	1
Platelets(10^9^/L)	235.55(59.91)	251.37(66.18)	0.99	0.324^a^	1
Albumin(g/L)	40.80(3.34)	40.63(2.62)	-0.24	0.815^a^	1
NLR	2.22(1.79)	2.88(3.06)	1.02	0.31^a^	1
NMR	7.77(2.34)	10.08(5.48)	1.578	0.115^b^	1
PLR	137.00(48.71)	138.96(75.01)	0.843	0.399^b^	1
NAR	0.09(0.02)	0.12(0.05)	2.81	0.004^b^	0.088
MLR	0.28(0.14)	0.27(0.16)	-0.19	0.848^a^	1

Data are presented as means (standard deviation). N is sample size

a Independent samples T-test

b Mann-Whitney U test

P Adjust: Using the Bonferroni correction to adjust P value (P value multiplied by 22, capped at1.000)

Abbreviation: SZN: non-agitated schizophrenia group; SZA: agitated schizophrenia group; IL: interleukin, IFN: interferon; TNF: tumor necrosis factor, NLR: neutrophil to lymphocyte ratio, NMR: neutrophil to monocyte ratio, PLR: platelet to lymphocyte ratio, NAR: neutrophil to albumin ratio, MLR: monocyte to lymphocyte ratio

### Correlation of peripheral inflammatory factors and differentially expressed miRNAs

**Fig. 4:**
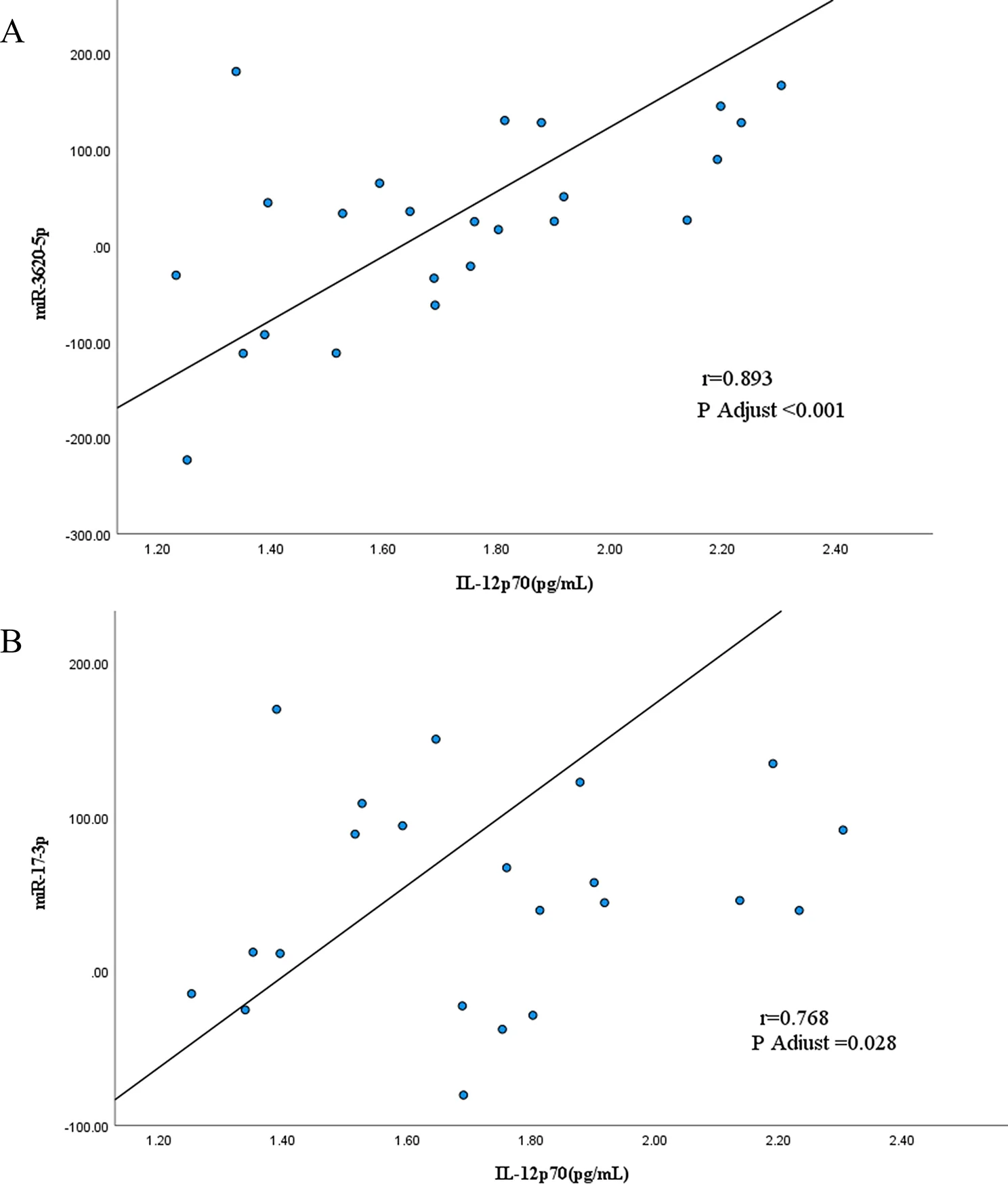
Associations between IL‑12p70 and plasma exosomal microRNAs in the SZA group. All scatter plots present partial correlation results after controlling for age, sex, BMI, chlorpromazine equivalent, and diazepam equivalent, plotted based on residuals plus mean. (**A**) Partial correlation between IL-12p70 and miR-3620-5p in SZA group. (**B**) Partial correlation between IL-12p70 and miR-17-3p. Using the Bonferroni correction to adjust P value. * significant at p Adjust < 0.05. ** significant at p Adjust < 0.005. ***significant at p Adjust < 0.001. Abbreviation: BMI: body mass index, IL: interleukin, miR: microRNA

Correlation of peripheral inflammatory factors and differentially expressed miRNAs was presented in Fig. 4. After Bonferroni‑corrected partial correlation analyses adjusting for age, sex, BMI, chlorpromazine equivalent, and diazepam equivalent, no significant correlations between 18 differentially expressed miRNAs and peripheral inflammatory factors were observed in SZN group. However, in SZA group, IL‑12p70 showed strong positive correlations with miR‑3620‑5p (Fig. 4A, *r* = 0.893, p Adjust < 0.001) and miR-17-3p (Fig. 4B, *r* = 0.768, p Adjust = 0.028). No other peripheral inflammatory factors exhibited a significant correlation with any differentially expressed miRNAs in SZA group. The heatmap also included correlations among inflammatory factors or among differentially expressed miRNAs themselves, which are not described here as they are beyond the focus of this study (see Supplementary Fig. 1).

### Predictive value of plasma exosomal miRNAs and peripheral inflammatory markers

A two-step approach was used to build the multivariable logistic regression model for agitation in patients with schizophrenia. First, forward stepwise logistic regression was performed among 18 differentially expressed miRNAs and 22 peripheral inflammatory factors, together with fixed covariates (age, sex, BMI, diazepam equivalent, and chlorpromazine equivalent), yielding PLR, NAR, and miR-543 as candidate variables. These candidates were then entered into the final multivariable logistic regression model together with fixed variables (Table 4). All continuous biomarkers were standardized to Z-scores.

All variance inflation factor (VIF) was below 1.5, indicating no serious multicollinearity. The model demonstrated good calibration (Hosmer–Lemeshow χ² = 4.433, df = 8, *p* = 0.816) and explained 42.5% of the variance in agitation status (Nagelkerke R² = 0.425). Among the biomarkers, NAR was significantly associated with increased odds of agitation (OR per SD increase = 3.778, 95% CI 1.459–9.781, Wald χ² = 7.501, *p* = 0.006). In contrast, miR-543 was significantly associated with reduced odds of agitation (OR per SD increase = 0.162, 95% CI: 0.032–0.806, Wald χ² = 4.943, *p* = 0.026). None of the other variables, including age, sex, medication doses, BMI, or PLR, reached statistical significance (all *p* > 0.05). These findings indicate that higher NAR levels and lower plasma exosomal miR-543 levels were independently associated with agitation in this sample.

**Fig. 5:**
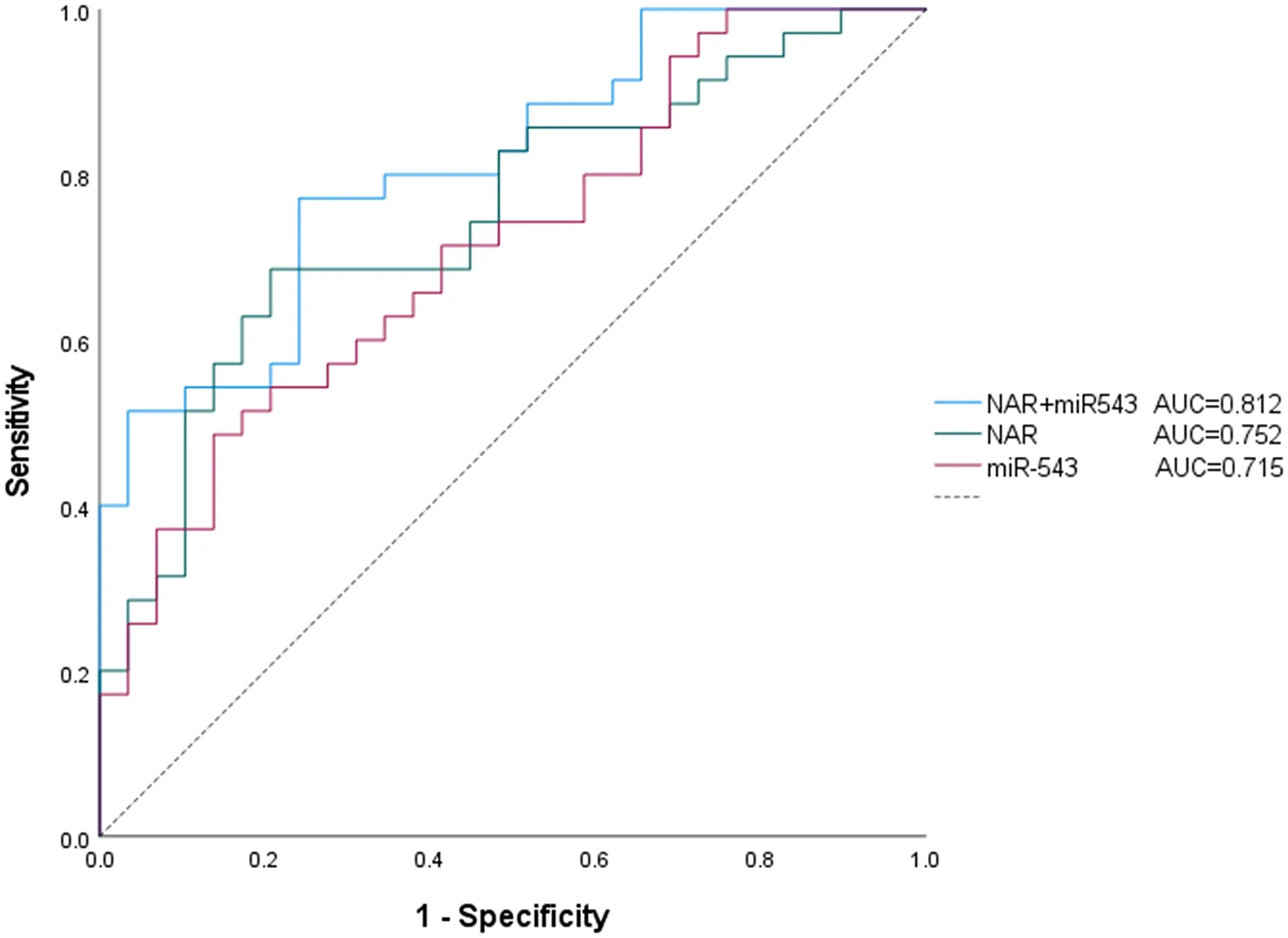
Receiver operating characteristic (ROC) curve of NAR, miR-543, and the combined model for agitation discrimination. Different colors denote the AUC for each miRNA and peripheral inflammatory factors. AUC: area under the curve, ROC: receiver operating characteristic, miRNA: microRNA, PLR: platelet to lymphocyte ratio, NAR: neutrophil to albumin ratio

We further built logistic regression models incorporating the fixed variables (age, sex, BMI, diazepam equivalent, chlorpromazine equivalent) together with each of the three biomarkers individually or in combination to discriminate between the SZA and SZN groups. The combined model including NAR and miR‑543 together with the fixed variables yielded an AUC of 0.812, while each single biomarker showed AUC of 0.752 for NAR and 0.715 for miR‑543 (Fig. 5).

**Table 4:** Logistic regression models for schizophrenia patients with agitation

Variable	B (SE)	Wald χ²	OR (95% CI)	*p* value	VIF
Fixed variables					
Age(years)	0.028(0.04)	0.488	1.028(0.951,1.112)	0.485	1.202
Male	1.194(0.721)	2.742	3.300(0.803,13.557)	0.098	1.312
Chlorpromazine Equivalent	0.000(0.002)	0.031	1.000(0.997,1.004)	0.861	1.254
Diazepam Equivalent	0.125(0.113)	1.225	1.133(0.908,1.412)	0.268	1.387
BMI, kg/m²	-0.107(0.08)	1.801	0.899(0.769,1.050)	0.180	1.355
Z-score standardized biomarkers					
PLR (per SD)	-0.522(0.424)	1.513	0.594(0.258,1.363)	0.219	1.340
NAR (per SD)	1.329(0.424)	7.501	3.778(1.459,9.781)	0.006	1.358
miR-543 (per SD)	-1.822(0.82)	4.943	0.162(0.032,0.806)	0.026	1.117
Model summary					
Nagelkerke R² 0.425					
Hosmer–Lemeshow χ², p value (df = 8) 4.433,0.816					

The model was adjusted for age, sex, BMI, diazepam equivalent, and chlorpromazine equivalent. All continuous biomarkers were standardized to Z‑scores (per SD increase)

Model summary: Nagelkerke R² = 0.425; Hosmer–Lemeshow test χ² (df = 8) = 4.433, *p* = 0.816

Abbreviation: SE: standard errors, OR: odds ratio, CI: confidence interval, VIF: variance inflation factor, PLR: platelet to lymphocyte ratio, NAR: neutrophil to albumin ratio, BMI: body mass index

### Validation of the performance of the NAR and miR-543 model

We examined variations in the performance of the two biomarkers and performed five-fold cross-validation, in which we iterated the following process five times: a logistic model was built using 80% of the participants, and its performance was assessed using the remaining 20%. The average AUC was 0.795 (95% CI 0.675–0.916). The sensitivity, specificity, and accuracy were 80.1 ± 16.5% (mean ± SD), 79.5 ± 26.1%, and 77.8 ± 8.9%, respectively, and the Youden index was 0.596 ± 0.117.

## Discussion

To our knowledge, this study is the first to investigate the plasma exosomal miRNA expression profiles and peripheral inflammatory factors between schizophrenia patients with agitation and patients without agitation. The key findings include that: (1) 18 differentially expressed miRNAs (15 upregulated and 3 downregulated) were identified in schizophrenia patients with agitation, with target genes enriched in pathways related to synaptic regulation and inflammation-associated signaling; (2) patients with agitation showed a trend toward elevated NAR levels, while no significant differences in other inflammatory factors were observed between patients with agitation and patients without agitation; (3) ROC analysis confirmed that the combination of NAR and plasma exosomal miR-543 showed moderate discriminative performance for agitated symptoms in schizophrenia. The diagnostic efficiency still remained stable after five-fold cross-validation. These findings provide preliminary evidence regarding plasma exosomal miRNA profiles in schizophrenia patients with agitation, and together with the higher peripheral NAR, suggest a potential multi-level biological pattern. This may offer new insights into the post‑transcriptional regulation and inflammation‑related pathways underlying agitation symptoms.

Pathway enrichment analysis of predicted target genes from the 18 differentially expressed miRNAs revealed distinct functional patterns that may contribute to the neurobiology of agitation in schizophrenia. Because pathway enrichment analysis is exploratory in nature, we focused our interpretation on pathways most closely related to inflammatory dysregulation and synaptic dysfunction, which were the primary biological processes underlying our study hypothesis. Targets of upregulated miRNAs showed GO enrichment primarily in intracellular signaling cascades and cytoskeletal remodeling, with KEGG pathway enrichment prominently in the MAPK signaling pathway and Ras signaling pathway. Although direct evidence in schizophrenia patients with agitation remains limited, previous studies have implicated the Ras-MAPK pathway in aggressive behavior across different species [43]. This interpretation is also consistent with reports that Ras-MAPK pathway alterations are associated with irritability-related phenotypes in RASopathies [44], despite not directly addressing agitation in schizophrenia. The Ras-MAPK signaling pathway is involved in various biological processes including synaptic development, dendritic spine remodeling, neuronal survival, and intracellular signal transduction. Studies using cellular and animal models suggest that the Ras-MAPK pathway is involved in regulating synaptic structure [45]. Postmortem brain tissue analyses of patients with schizophrenia also indicated that dysregulation of Ras-MAPK signaling pathway can lead to synaptic structural abnormalities and reduced neural plasticity [46, 47].Combined with the GO analysis results from our study, the upregulated miRNA targets were significantly associated with synaptic structural component, including the synaptic membrane, distal axon, and neuronal cell body, as well as molecular functions such as actin binding and phospholipid binding. This finding may suggest alterations in synaptic regulation associated with agitation symptoms in schizophrenia. In addition to synaptic structure, the Ras-MAPK pathway may also be involved in inflammatory processes. The Ras-MAPK signaling pathway can regulate the expression of proinflammatory factors (IL-1β, IL-6, TNF-α), and has been implicated in neuroinflammatory processes within the central nervous system [48]. This subsequently activates microglia, causing dysfunction in neural circuits in the amygdala. As the core brain region for emotional regulation, the dysfunction of neural circuits leads to impaired control over emotions and behavior, increasing the risk of aggressive behavior [49]. Besides the Ras-MAPK signaling pathway, upregulated miRNAs were also enriched in cholinergic synapses. Consistent with prior findings, positron emission tomography (PET) and single photon emission computed tomography (SPECT) scans of SZ patients have revealed that lower expression of β2 nicotinic acetylcholine receptors (nAChRs) in the striatum correlated with more severe agitation symptoms [50]. Meanwhile, cholinergic synapse agonists, such as nicotine, effectively reduce agitation scores when used via nicotine patches to acute-phase schizophrenia patients. Upon activation by nicotine, cholinergic synapses negatively regulate striatal dopamine release via M1/M4 muscarinic acetylcholine receptor (mAChR), thereby relieving agitation symptoms [51]. These findings suggest that cholinergic signaling may be involved in the modulation of agitation. Furthermore, the enrichment in efferocytosis-related pathways suggests that impaired clearance of apoptotic material may sustain inflammatory signaling while simultaneously intersecting with cytoskeletal reorganization. Pathway enrichment analysis of the predicted target genes of downregulated miRNAs also showed notable dysregulation in the glutamatergic synaptic pathway. Similarly, autopsy and imaging studies revealed dysregulation of glutamatergic synapses across multiple brain regions in schizophrenia patients. This dysregulation included increased glutamatergic synaptic density in the striatum and decreased density in the prefrontal cortex, leading to impaired control over emotions and behavior [12, 52]. Moreover, evidence indicated that inflammatory signaling pathways, like Ras-MAPK and efferocytosis signaling pathways, can modulate synaptic plasticity and neurotransmitter systems, suggesting a potential link between immune activation and synaptic dysfunction [53]. These findings suggest that alterations in synaptic regulation and inflammation-associated signaling may be associated with agitation symptoms in schizophrenia. It should be noted that since this study performed pathway analysis based on plasma exosomal miRNAs, the results cannot yet clearly reflect the specific brain region localization of these alterations within the central nervous system.

The mildly elevated NAR in the SZA group may indicate subtle alterations in peripheral immune status. However, this finding should be interpreted cautiously because this difference did not remain significant after strict multiple-comparison correction. Previous studies have reported elevated NAR in patients with schizophrenia across different disease stages, suggesting that this marker may reflect relatively stable changes in peripheral immune-related processes [54]. In contrast, circulating cytokines are more dynamic inflammatory mediators and can be influenced by disease stage, antipsychotic treatment, circadian variation, and other biological factors [24, 25]. Although several pro-inflammatory cytokines, including IL-1β, IL-6, and IFN-γ, have been reported to increase during the acute phase of schizophrenia [55, 56], our sample mainly consisted of patients with chronic illness and a history of relapse (mean disease duration: 8.67 years), and antipsychotic exposure may further attenuate peripheral cytokine alterations [55, 57]. Therefore, the absence of significant cytokine differences between the SZA and SZN groups does not necessarily exclude subtle immune-related alterations. Nevertheless, larger longitudinal studies are required to further clarify the relationship between peripheral immune markers and agitation symptoms in schizophrenia.

Correlation analysis indicated that the relationship between peripheral inflammatory activity and differentially expressed plasma exosomal miRNAs was inconsistent across the groups. After Bonferroni correction and adjustment for gender, age, BMI, chlorpromazine equivalent and diazepam equivalent, no significant associations were observed between the 18 differentially expressed miRNAs and peripheral inflammatory factors in the SZN group. In contrast, in the SZA group, IL-12p70 showed a significant positive correlation with miR-3620-5p and miR-17-3p, suggesting a possible association between plasma exosomal miRNA alterations and the IL-12-related immune axis in the SZA group. As a key driver of Th1 immune polarization, IL-12p70 promotes IFN-γ secretion and enhances cytotoxic immune responses [58, 59]. The correlation between IL-12p70 and miR-17-3p is particularly noteworthy. MiR-17-3p originates from the miR-17-92 gene cluster, which is known to regulate cell proliferation and inflammation regulation [60, 61]. The correlation between miR-17-3p and IL-12p70 may reflect either a shared upstream inflammatory response or a preliminary regulatory relationship that warrants further experimental validation. At present, the functional role of miR-17-3p has been demonstrated only in cell and mouse studies, and there are no schizophrenia animal models available. Further experiments are needed to investigate the regulatory relationship between miR-17-3p and IL-12p70. The function of miR-3620-5p remains unclear. But the correlation coefficient between miR-3620-5p and IL-12p70 is as high as 0.893, suggesting that it may be a candidate molecule worthy of further study in agitation-related biology. Nevertheless, further research is needed to identify its target genes and functional pathways. It is worth noting that, apart from IL-12p70, no other inflammatory factors showed significant correlations with the differentially expressed miRNAs in the SZA group, which suggests a potentially selective association. The heatmaps also visualize the correlations among inflammatory factors and among miRNAs, which may provide clues for future mechanistic studies. Future studies involving larger sample sizes of schizophrenia patients with agitation are needed, incorporating longitudinal follow-up and in vitro functional experiments, to verify whether the miR-3620-5p/miR-17-3p–IL-12p70 axis is involved in the pathogenesis of agitation.

Using a forward stepwise logistic regression approach, we first screened 18 differentially expressed miRNAs and 22 peripheral inflammatory factors, and PLR, NAR, and miR‑543 were retained as the selected variables. These three biomarkers were then entered into a multivariable logistic regression model together with fixed variables (age, sex, BMI, diazepam equivalent, and chlorpromazine equivalent). In this adjusted model, NAR was significantly associated with increased odds of agitation, while miR‑543 was associated with reduced odds. However, PLR and all fixed variables did not reach statistical significance. The model demonstrated good calibration and explained 42.5% of the variance in agitation status. The combined model incorporating NAR, and miR‑543 together with fixed variables yielded an AUC of 0.812, showing improved discriminative performance compared with the single-marker models (NAR AUC = 0.752, miR‑543 AUC = 0.715). Five-fold cross-validation suggested that the predictive performance of the model remained relatively stable. The average AUC of 0.795 (95% CI 0.675–0.916) indicated that the model still retained moderate predictive performance. MiR-543 has been experimentally shown to regulate the inflammation, which can suppress the expression of Kruppel-like factor 6(KLF6), blocking the transcriptional expression of its downstream proinflammatory factors (TNF-α, IL-1β, IL-6, etc.) [62]. As discussed above, elevated NAR levels indicate the presence of a dysregulation in inflammation [63]. Downregulation of miR-543 together with the trend toward higher NAR may reflect subtle immune dysregulation in the SZA group. In summary, through a systematic forward stepwise screening and multivariable modeling strategy, plasma exosomal miR‑543 and peripheral inflammatory factor NAR emerge as potentially useful and easily accessible peripheral biomarkers associated with agitation in schizophrenia.

### Limitations

This study had some limitations. First, the sample size was relatively small. Although we additionally used alternative statistical methods (the Bonferroni correction and the Benjamini–Hochberg correction) to obtain reliable and accurate results. A retrospective power analysis showed that among the miRNAs identified through correction, the miRNA with the smallest absolute log₂ fold change had a Hedges’ g of 0.357. Given the current sample size and a two-sided α = 0.05, the statistical power to detect this effect was only 29.5%. Sensitivity analysis further showed that an effect size of d = 0.715 is required to achieve 80% power. Therefore, although this study has sufficient statistical power to detect moderate-to-large miRNAs differences, it lacks sufficient power to detect more subtle but potentially biologically significant changes. Secondly, although five-fold cross-validation was performed, there is still a lack of an independent external validation cohort. Future studies with larger sample sizes and independent external validation are needed to confirm robustness and clinical value of plasma exosomal miRNAs. Third, although exosomes are capable of crossing the BBB, plasma exosomal miRNAs still cannot fully reflect the central inflammatory activity or identify the specific brain regions involved. Future research should explore the analysis of exosomes derived from specific cell sources, such as neuron-derived or glial cell-derived exosomes. Fourth, although patients with tobacco or alcohol use were excluded and statistical adjustment was performed for BMI, chlorpromazine equivalent, and diazepam equivalent, residual confounding related to medication exposure cannot be completely ruled out. Because patients with agitation frequently experience poor insight, altered consciousness, or reduced compliance during acute episodes, reliable assessment of pre-admission medication history was difficult. Therefore, future studies involving first-episode drug-free patients are warranted. Fifth, although antipsychotic medications were statistically analyzed using chlorpromazine equivalent for both groups, the long-term effects of antipsychotic medication on plasma exosomal miRNAs and peripheral inflammatory factors cannot be ruled out. Sixth, this study focused exclusively on acute agitation assessed within 24 h of hospital admission. Due to the cross‑sectional design, we cannot determine whether the observed molecular alterations are specific to the acute agitated state or represent a persistent trait. Longitudinal follow‑up studies are needed to assess whether these changes normalize after symptom resolution or remain stable over time. Finally, agitation in schizophrenia frequently co-occurs with more severe positive symptoms, making it difficult to fully disentangle specific molecular alterations from those associated with broader psychopathological severity. Therefore, the identified miRNAs may reflect overlapping biological mechanisms related to heightened psychotic symptom burden rather than a completely independent molecular signature specific to agitation symptoms.

## Conclusion

In summary, the findings indicate that schizophrenia patients with agitation symptoms exhibit alterations in plasma exosomal miRNA expression profiles, providing novel insights into the potential involvement of synaptic dysfunction and inflammation-associated signaling in the pathophysiology of agitation. Compared with the SZN group, the SZA group exhibited 18 differentially expressed plasma exosomal miRNAs. Functional enrichment analyses indicated that the predicted target genes of upregulated miRNAs were mainly enriched in the Ras-MAPK signaling pathway, efferocytosis, and cholinergic synapse-related pathways, whereas the predicted target genes of downregulated miRNAs were primarily enriched in glutamatergic synapse-related pathways. From the perspective of post-transcriptional gene regulation, these alterations may suggest that schizophrenia patients with agitation have changes in synaptic dysregulation and inflammation-associated signaling. Concurrently, the observed trend toward increased NAR further supported this interpretation, although these findings were preliminary. Furthermore, a diagnostic model combining plasma exosomal miR-543 and NAR showed moderate and stable differentiation efficacy for agitation symptoms in SZ patients, with a comprehensive AUC of 0.812 and with a 5-fold cross-validation mean AUC of 0.795, suggesting the potential of plasma exosomal microRNAs as candidate non-invasive biomarkers for agitation assessment. Future studies should include larger independent cohorts for external validation and should clarify the regulatory mechanisms of differentially expressed miRNAs through cell and animal experiments, in order to explore the pathophysiological processes underlying agitated symptoms in schizophrenia.

## Supplementary Information

Below is the link to the electronic supplementary material.

Supplementary Material 1

Supplementary Material 2

Supplementary Material 3

## Data Availability

The datasets used during the current study are available from the corresponding author on reasonable request.

## Abbreviations

SZ: Schizophrenia
miRNAs: microRNAs
SZA: Agitated patients with Schizophrenia
SZN: Non-agitated patients with Schizophrenia
ROC: Receiver operating characteristic
NAR: Neutrophil-to-albumin ratio
PSD95: Postsynaptic density protein 95
IL-6: Interleukin-6
TNF-α: Tumor necrosis factor-α
CRP: C-reactive protein
BBB: Blood-brain barrier
Qalb: Albumin quotient
CSF: Cerebrospinal fluid
MAP1B: Microtubule-associated protein 1B
DTNBP1: Dystrobrevin binding protein 1
DSM-5: Diagnostic and Statistical Manual of Mental Disorders, fifth edition
PANSS: Positive and negative syndrome scale
PANSS-EC: PANSS excitatory component
ELISA: Enzyme-linked immunosorbent assay
NLR: Neutrophil to lymphocyte ratio
NMR: Neutrophil to monocyte ratio
PLR: Platelet to lymphocyte ratio
MLR: Monocyte to lymphocyte ratio
TEM: Transmission electron microscopy
NTA: Nanoparticle tracking analysis
BP: Biological process
MF: Molecular function
CC: Cellular component
GO: Gene Ontology
KEGG: Kyoto Encyclopedia of Genes and Genomes
LR: Logistic regression
FC: Fold change
BMI: Body mass index
AUC: Area under the curve
FDR: False discovery rate
SD: Standard deviation
IQR: Interquartile range
CI: Confidence interval
OR: Odds ratio
PET: Positron emission tomography
SPECT: Single photon emission computed tomography
VIF: Variance inflation factor
df: Degree of freedom
nAchRs: nicotinic acetylcholine receptors
mAchR: muscarinic acetylcholine receptor
KLF6: Kruppel-like factor 6
